# Inflammatory proteins as acute biomarkers of post-traumatic epilepsy

**DOI:** 10.3389/fneur.2025.1720112

**Published:** 2025-11-20

**Authors:** Hild Flatmark Sødal, Silvia Balosso, Annamaria Vezzani, Laura Pasetto, Valentina Bonetto, Stefano Fabrizio Columbro, Robert McCarter, Eirik Helseth, Erik Taubøll, Pavel Klein

**Affiliations:** 1 Institute of Clinical Medicine, University of Oslo, Oslo, Norway; 2 Department of Neurology, Oslo University Hospital, Oslo, Norway; 3 Department of Acute Brain and Cardiovascular Injury, Istituto di Ricerche Farmacologiche Mario Negri IRCCS, Milan, Italy; 4 Department of Neuroscience, Istituto di Ricerche Farmacologiche Mario Negri IRCCS, Milan, Italy; 5 Department of Biostatistics and Research Design, Children's National Medical Center, Washington, DC, United States; 6 Department of Neurosurgery, Oslo University Hospital, Oslo, Norway; 7 Mid-Atlantic Epilepsy and Sleep Center, Bethesda, MD, United States

**Keywords:** epilepsy, neuroinflammation, HMGB1, MMP-9, interleukins, seizures

## Abstract

**Objective:**

To evaluate the potential of targeted inflammatory proteins high mobility group box 1 (HMGB1), matrix metalloproteinase 9 (MMP-9) and interleukins (IL)-6, IL-8 and IL-10 as early biomarkers for post-traumatic epilepsy (PTE) prediction.

**Methods:**

In this prospective, international study, adult patients with traumatic brain injury (TBI) and an anticipated high risk of PTE based on radiological and clinical findings were recruited from Level 1 trauma centers in the USA and Europe. Blood was collected on days 2 and 4 post-TBI. Patients were followed clinically for 24 months for PTE development. Serum levels of the inflammatory markers were assessed using commercially available ELISA and AlphaLISA kits and compared between patients who did and did not develop PTE, and between PTE and a subgroup of non-PTE patients matched for age, sex, and Glasgow Coma Scale using non-parametric tests.

**Results:**

We found no statistically significant differences in serum levels of the inflammatory markers between PTE patients (*n* = 13) and non-PTE patients (*n* = 73), neither at each timepoint nor in the change from day 2 to day 4. Exploring temporal changes within each group, we found a significant decrease in IL-6 level between the two timepoints in the total and matched non-PTE groups, but not in the PTE group. MMP-9 level decreased in both the PTE and the matched non-PTE groups, but not in the total non-PTE group.

**Significance:**

Based on our findings, serum levels of HMGB1, MMP-9, IL-6, IL-8 and IL-10 measured at early time points after TBI may not serve as sensitive biomarkers of PTE. However, a faster decline in IL-6 levels in the non-PTE groups suggests a more rapid resolution of inflammation among patients who do not develop PTE, supporting the role of neuroinflammatory mechanisms in epileptogenesis. The potential of IL-6’s temporal profile as a biomarker of PTE warrants further exploration.

## 1 Introduction

Epilepsy is a well-known complication of traumatic brain injury (TBI), associated with increased mortality, poor functional outcome and reduced quality of life (1–5). Post-traumatic epilepsy (PTE), defined as unprovoked seizures occurring >7 days after TBI, causes approximately 5% of all epilepsy (6).

The clinical manifestation of PTE is preceded by a latent period following TBI, where cellular and molecular changes occur that eventually result in increased seizure susceptibility and epilepsy (7). Attempts to prevent PTE have so far been unsuccessful (8). However, no phase 3 clinical trials of prevention have been initiated in the last 25 years, in spite of a large number of drugs that have shown to have antiepileptogenic effects in animal models (9, 10). Biomarkers of PTE prediction are essential for identifying patients at high risk of PTE who could benefit from therapeutic interventions and enrich the patient population in future preventive trials (10). Numerous animal studies and retrospective human studies have identified potential biomarkers of PTE prediction, but they have not been previously evaluated in prospective human studies and their biological significance and clinical utility are still not established (11, 12).

Neuroinflammation is considered one of the main candidate mechanisms of epileptogenesis after acute brain injury (6, 13). Inflammatory pathways may serve as targets for novel treatments to stop the development and progression of epilepsy, and as potential biomarkers for epileptogenesis (14).

Cytokines, including interleukins and high mobility group box 1 (HMGB1) are released rapidly by activated neurons and reactive glia after brain injury, and drive the neuroinflammatory cascade, acting as signaling molecules with neuromodulatory properties (15, 16). They contribute to both repair mechanisms and pathophysiology after TBI (17). TBI leads to disruption of the blood–brain barrier (BBB), allowing inflammatory molecules to leak into the peripheral circulation, potentially contributing to systemic inflammation which in turn could exacerbate the pathogenesis of TBI (18). Cytokines have been associated with development of seizures in patients and in animal studies (14). In particular, HMGB1 is a key mediator of neuroinflammation involved in epileptogenesis in animal models of acquired epilepsy (19, 20). Elevated blood levels of HMGB1 have been measured in patients with drug-resistant compared to drug-sensitive epilepsy and healthy controls (21, 22). IL-6, together with IL-8, was associated with MRI signal abnormalities in children with febrile status epilepticus at higher risk of developing epilepsy (23). IL-10, an anti-inflammatory cytokine crucially involved in the resolution of the inflammatory response, may offer protective effects by preventing prolonged inflammation and by enhancing inhibitory GABA_A_ receptor currents (24, 25).

Another inflammatory protein of interest is matrix metalloproteinase 9 (MMP-9), a proteolytic enzyme involved in extracellular matrix remodeling, BBB breakdown and neuroinflammation after TBI (26). The involvement of MMP-9 in epileptogenesis in patients and rodents has been previously documented (27). In an animal model of TBI, mice overexpressing MMP-9 had an increased risk of PTE, while the opposite was observed in MMP-9 knockout mice, indicating a functional role of MMP-9 in post-TBI epileptogenesis (28).

Based on this evidence, the primary objective of this study was to evaluate the potential value of the inflammatory proteins HMGB1, MMP-9 and IL-6, IL-8 and the anti-inflammatory cytokine IL-10 as early biomarkers of PTE. We hypothesized that serum levels of the inflammatory proteins would be higher, whereas those of IL-10 would be lower, in TBI patients who developed epilepsy compared to those who did not, reflecting the development of an excessive inflammatory response. Secondarily, we explored the relationship between the inflammatory proteins and long-term mortality.

## 2 Materials and methods

### 2.1 Study design and participants

This study is part of the project “Genetic and protein biomarkers of post-traumatic epilepsy to improve prediction of PTE: a prospective study in an enriched patient population.” From July 2020 to December 2021, patients with TBI and an anticipated risk of PTE of approximately 30%, based on data from prior epidemiological, observational, and placebo-controlled studies (1, 29, 30), were recruited at ten Level 1 trauma centers, seven in the USA and three in Europe. Written informed consent was obtained from all patients or legal representatives at enrollment.

Eligible patients were those aged 18–75 with acute TBI, Glasgow Coma Scale (GCS) >3 and one or more of the following TBI characteristics: (1) subdural hematoma (SDH) requiring acute surgery, (2) combined SDH and cerebral hemorrhage or contusion, (3) multiple cerebral contusions, (4) penetrating injury and (5) depressed skull fracture. Exclusion criteria were GCS of 3, prior epilepsy or seizures within 3 years of enrollment, use of an anti-seizure medication (ASM) within 1 month prior to TBI, previous acute CNS insult within 2 years of enrollment, progressive CNS disorder, unstable medical condition not related to the trauma, active drug or alcohol dependency and moderate to severe intellectual disability.

The first clinical follow up visit occurred at 3 months after TBI. Participants who died or were lost to follow-up before the first clinical follow up visit were excluded, because it was not possible to evaluate the presence of PTE.

All patients received standard TBI treatment according to guidelines (31). At the US sites, this included seizure prophylaxis with levetiracetam or phenytoin during the first week after TBI. At the European sites, ASM prophylaxis was used sporadically.

### 2.2 Data collection, evaluations, and definitions

Data were collected and managed using the REDCap electronic data capture tools hosted at Children’s National Medical Center, Washington, DC (32). Demographics and clinical data were retrieved from medical records at enrollment. CT brain scan findings were recorded from reports by the study investigators. Information regarding acute treatment including neurosurgical interventions, and occurrence of clinical or electrographic seizures during hospital stay was registered. Seizures occurring within the first week after TBI were classified as early post-traumatic seizures (EPTS). TBI with any intracranial hemorrhage was defined as severe TBI. GCS was divided into three categories: 4–8, 9–12 and 13–15. The GCS recorded was the lowest value registered prior to enrollment, except when GCS 3 occurred transiently. The term multitrauma was applied if patients had concomitant non-CNS injury other than contusions and skin lacerations.

Follow-up was done by telephone interviews or face-to-face visits at 3, 6, 12, 18 and 24 months after TBI. Evaluations included an interview with patients or their caregivers, assessment of intercurrent illnesses and medications and a structured seizure questionnaire. Patients with possible or suspected seizures were evaluated by the site’s epileptologist, either by telephone or face to face. PTE was defined as one unprovoked seizure occurring >7 days after TBI. If information about seizures was inconsistent or seizures were judged to be non-epileptic, the patient was classified as non-PTE. Long-term mortality was defined as mortality >3 months after TBI.

### 2.3 Blood sampling and protein analysis

Blood was collected in serum tubes with clot activator on day 2 (24–48 h) and day 4 (72–96 h) after TBI. The clot was removed after centrifugation (2,500 rpm for 10 min) at 4 °C. Serum was collected, aliquoted and stored at −80 °C until assay. All sites followed the same protocol for sample collection and handling.

#### 2.3.1 HMGB1 assay

Total HMGB1 levels were determined by commercially available ELISA for the human protein (#ST51011, IBL International) according to the manufacturer’s guidelines, as described previously (33). Since HMGB1 can be released from damaged erythrocytes and haemoglobin interferes with ELISA assay, samples that appeared visibly red were classified as hemolytic and were not included in the measurements.

In brief, thawed samples were diluted in sample diluent (dilution 1:2). Then, 100 μL of diluted samples were pipetted into the wells of the microtiter plate in duplicate and incubated overnight at 37 °C for 20 h. Plates were washed 6 times in washing buffer (300 μL/well). Detection antibody solution was added (100 μL/well) for 2 h at 25 °C. Following washing steps, substrate solution was added (100 μL/well) and incubated at room temperature protected from light with foil seal for 30 min. After incubation, the sample plate was read at 450 nm. Data were fitted to the standard curve (0–10 ng/mL).

#### 2.3.2 MMP-9, IL-6, IL-8, and IL-10 AlphaLISA assay

The levels of MMP-9, IL-6, IL-8, and IL-10 were quantified using an AlphaLISA kit for the human protein (#AL3138, #AL223, #AL224, #AL218 Revvity, respectively), as previously described (34). In brief, the thawed samples were diluted in accordance with the manufacturer’s instructions using the provided sample diluent. Subsequently, 5 μL of the diluted samples, anti-analyte acceptor beads, and biotinylated anti-analyte antibody were added to the wells of the microtiter plate in duplicate, and the mixture was incubated for 60 min. Streptavidin-donor beads substrate solution was then added and incubated for 90 min. Finally, the AlphaLISA signal was quantified using the Ensight Multimode Plate Reader (Revvity). Data were plotted against the respective standard curves.

### 2.4 Statistical analysis

Categorical variables are presented as frequencies with percentages, and continuous variables as medians with interquartile ranges (IQR). Differences between the PTE and non-PTE groups were tested using Fischer’s exact test for categorical variables and Mann–Whitney U test for continuous variables, including biomarker levels. The Wilcoxon signed-rank test was used to evaluate changes within groups over time by comparing day 2 and 4 levels for the PTE and non-PTE groups separately. To maintain the integrity of the dataset, we included all values in the analyses. In the figures, outliers were removed for improved visualization. Differences were considered statistically significant at a *p*-value of <0.05.

Due to the small number of PTE cases and the heterogeneous nature of the cohort, we performed a secondary analysis comparing the PTE group with a subcohort of non-PTE patients, matched 2:1 for age, sex, and GCS. The subcohort was selected from those with at least 18 months of follow-up to reduce the risk of misclassifying PTE status in those lost to follow-up. Analyses were performed using Stata version 18.0.

## 3 Results

### 3.1 Characteristics of the study population

One hundred and ten patients were enrolled; the family of one patient withdrew their consent immediately after consenting. Of the remaining 109 patients, 23 (21%) died (*n* = 11) or were lost to follow-up (*n* = 12) during the first 3 months, resulting in a study cohort of *n* = 86. Of these, 56% were enrolled in USA and 44% in Europe. There were no differences in sex (*p* = 1.00), age (*p* = 0.17) or GCS (*p* = 0.48) between those who died or were lost to follow-up and the final study cohort. Demographic and injury characteristics are summarized in Table 1. Median (IQR) age was 48 (30, 62) years and 67 (78%) were male. Except for one patient with only depressed skull fracture, all patients had intracranial hemorrhage evident on head CT scan. Multitrauma was present in 28%, with extremity fracture(s) being the most frequent additional injury. Forty-six patients (53%) received early seizure prophylaxis with levetiracetam and four (4.7%) with phenytoin. Neurosurgery (craniotomy, craniectomy or cranioplasty) was performed in 33 (38%). Eight patients (9.3%) had EPTS, all occurring between 0 and 5 days after TBI.

**Table 1:** Patient demographic and clinical characteristics.

Characteristic	All patients (*n* = 86)	PTE patients (*n* = 13)	Non-PTE patients (*n* = 73)	*p*-value
Age, years (median, IQR)	48 (30, 62)	42 (24, 51)	51 (32, 63)	0.15
Sex, n (%)				0.72
Male	67 (78%)	11 (85%)	56 (78%)	
Female	19 (22%)	2 (15%)	17 (23%)	
Comorbidity, n (%)				0.37
0–1	47 (55%)	9 (69%)	38 (52%)	
≥2	39 (45%)	4 (31%)	35 (48%)	
BMI*, n (%)				0.052
≤25 kg/m^2^	28 (34%)	1 (7.7%)	27 (39%)	
>25 kg/m^2^	55 (66%)	12 (92%)	43 (61%)	
GCS, n (%)				0.59
4–8	30 (35%)	6 (46%)	24 (33%)	
9–12	19 (22%)	3 (23%)	16 (22%)	
13–15	37 (43%)	4 (31%)	33 (45%)	
Injury type**, n (%)				
SDH requiring surgery	16 (22%)	5 (38%)	14 (19%)	0.15
SDH + cerebral hemorrhage	55 (64%)	9 (69%)	46 (63%)	0.76
Multiple contusions	32 (37%)	6 (46%)	26 (36%)	0.54
Depressed skull fracture	11 (13%)	2 (15%)	9 (12%)	0.67
Penetrating injury	6 (7%)	3 (23%)	3 (4.1%)	**0.04**
Multitrauma	24 (28%)	7 (54%)	17 (23%)	**0.04**

PTE, post-traumatic epilepsy; IQR, interquartile range; BMI, Body Mass Index; GCS, Glasgow Coma Scale; SDH, subdural hemorrhage; ICH, intracerebral hemorrhage. Significant values are presented in bold; *3 missing; **Not mutually exclusive, several patients experienced more than one type of injury.

During follow-up, six patients (7.0%) died and 10 (12%) were lost to follow-up before developing PTE or completing 24 months of follow-up. Median (IQR) follow up was 24 (15, 24) months. A total of 13 patients (15%) developed PTE. Of these, 85% had their first late seizure within 12 months following TBI. Two (15%) had EPTS, compared to six (8.2%) of the non-PTE patients (*p* = 0.35). All patients who developed PTE had frontal lobe injury. Ten patients (77%) had additional temporal injury, five (38%) had parietal injury, and two (15%) had occipital injury. The incidence of PTE was higher in patients with penetrating injury compared to those without penetrating injury (50% vs. 12.5%, *p* = 0.04). Of the patients undergoing neurosurgery (craniotomy, craniectomy or cranioplasty), PTE incidence was 21% versus 11% in those who did not undergo such treatments (*p* = 0.23). The median age at the time of TBI was lower in the PTE group than in the non-PTE group, but the difference was not statistically significant. In addition to penetrating injury, multitrauma was more frequent in PTE than non-PTE patients (*p* = 0.04).

The matched non-PTE subgroup (*n* = 26) had a median (IQR) age of 41 (23, 56) years and 85% were male. Median (IQR) GCS was 9 (7, 13) compared to 10 (7, 13) in the PTE group.

#### 3.1.1 Serum samples

Eighty one (94%) of the patients had a complete set of blood samples, the remaining had either day 2 (*n* = 2 PTE and 2 non-PTE) or day 4 sample (*n* = 1, non-PTE). A small number of samples were not usable due to hemolysis (HMGB1 analysis; *n* = 9, 2 PTE and 7 non-PTE) or insufficient volume (interleukin analyses; *n* = 11, non-PTE).

### 3.2 HMGB1, MMP-9 and interleukin analysis

Serum levels of HMGB1, MMP-9, IL-6 and IL-8 for the whole cohort are presented in Table 2. IL-10 was not detectable in 94% of the samples and was therefore excluded from further analysis. There was a statistically significant decrease in median levels of MMP-9 and IL-6 from day 2 to day 4 (*p* = 0.001 and *p* < 0.001, respectively). The 15% decrease in HMGB1 and 9% increase in IL-8 were not statistically significant.

**Table 2:** Mean (SD) and median (IQR) serum levels of the inflammatory proteins at day 2 and day 4 after TBI for the whole cohort (PTE and non-PTE patients).

Biomarker and time	Samples (*n*)	Mean (SD)	Median (IQR)
HMGB1 day 2	81	5.69 (6.03)	3.75 (1.92, 6.83)
HMGB1 day 4	77	4.31 (3.73)	3.18 (1.50, 5.98)
MMP-9 day 2	85	274.5 (131.3)	246.3 (172.1, 387.0)
MMP-9 day 4	82	237.3 (114.7)	216.8 (150.3, 318.9)
IL-6 day 2	77	16.0 (28.8)	6.77 (2.49, 12.6)
IL-6 day 4	79	7.69 (14.4)	2.57 (0, 7.73)
IL-8 day 2	77	2.31 (3.0)	1.26 (0.54, 2.65)
IL-8 day 4	79	3.59 (10.5)	1.37 (0.67, 2.58)

HMGB1 and MMP-9 in ng/mL, IL-6 and IL-8 in pg/mL. Samples (*n*) refers to the available number of samples for each biomarker analysis. HMGB1, High Mobility Group Box 1; MMP-9, Matrix metalloproteinase 9; IL-6, Interleukin-6; IL-8, Interleukin-8.

#### 3.2.1 HMGB1, MMP-9, IL-6 and IL-8 levels and PTE

Comparing the PTE group (*n* = 13) and the non-PTE group (*n* = 73), there were no statistically significant differences in the median levels of any of the proteins at the two time points (Table 3), nor in the change in levels from day 2 to day 4.

**Table 3:** Median (IQR) serum levels of the inflammatory proteins by PTE and death.

	HMGB1 day 2	HMGB1 day 4	MMP-9 day 2	MMP-9 day 4	IL-6 day 2	IL-6 day 4	IL-8 day 2	IL-8 day 4
PTE status								
PTE (*n* = 13)	3.90 (2.53, 4.26)	3.29 (1.50, 4.45)	269.5 (193.1, 385.7)	163.4 (149.0, 222.2)	7.00 (6.72, 14.6)	5.60 (1.74, 12.7)	1.16 (0.90, 4.46)	1.56 (1.02, 2.33)
Non-PTE (*n* = 73)	3.17 (1.87, 7.40)	3.16 (1.44, 5.99)	242.2 (168.2, 389.9)	231.2 (150.3, 336.9)	6.07 (0, 12.2)	2.57 (0, 6.68)	1.37 (0.48, 2.42)	1.33 (0.44, 2.59)
*p*-value	0.98	0.95	0.89	0.22	0.18	0.17	0.41	0.36
Death								
Yes (*n* = 6)	4.06 (1.45, 6.45)	3.74 (1.71, 6.89)	207.2 (81.9, 393.4)	212.0 (166.0, 266.7)	22.5 (3.49, 51.9)	12.4 (6.72, 26.5)	3.50 (1.38, 4.09)	1.09 (0, 5.31)
No (*n* = 80)	3.75 (1.92, 6.98)	3.14 (1.39, 5.98)	248.7 (172.1, 387.0)	218.6 (149.6, 321.8)	6.72 (0, 12.1)	2.25 (0, 6.59)	1.22 (0.53, 2.36)	1.48 (0.70, 2.58)
*p*-value	0.80	0.51	0.41	0.70	0.15	**0.03**	0.13	0.83

HMGB1 and MMP-9 in ng/mL, IL-6 and IL-8 in pg/mL. Significant values are in bold. PTE, post-traumatic epilepsy; HMGB1, High Mobility Group Box 1; MMP-9, Matrix metalloproteinase 9; IL-6, Interleukin-6; IL-8, Interleukin-8.

**Figure 1:**
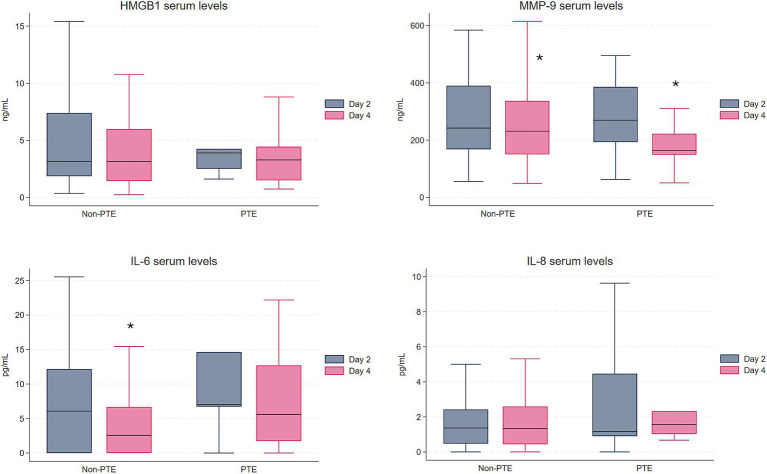
Serum levels of HMGB1, MMP-9, IL-6, and IL-8 in the PTE group (*n* = 13) and the non-PTE group (*n* = 73). To enhance visualization, outliers are not displayed; they were included in all analyses. *Indicates a statistically significant change between day 2 and day 4 within the group. HMGB1 and MMP-9 in ng/mL, IL-6 and IL-8 in pg/mL. HMGB1, High Mobility Group Box 1; MMP-9, Matrix metalloproteinase 9; IL-6, Interleukin-6; IL-8, Interleukin-8.

Within group analyses revealed a statistically significant decrease in IL-6 from day 2 to day 4 in the non-PTE group (*p* < 0.001), whereas there was no decrease in the PTE group (Figure 1). MMP-9 decreased over time similarly in both the PTE (*p* = 0.02) and non-PTE groups (*p* = 0.009). For both HMGB1 and IL-8, changes in serum levels between the two time points were not statistically significant within either group.

#### 3.2.2 Subgroup analysis, matched cohort

**Figure 2:**
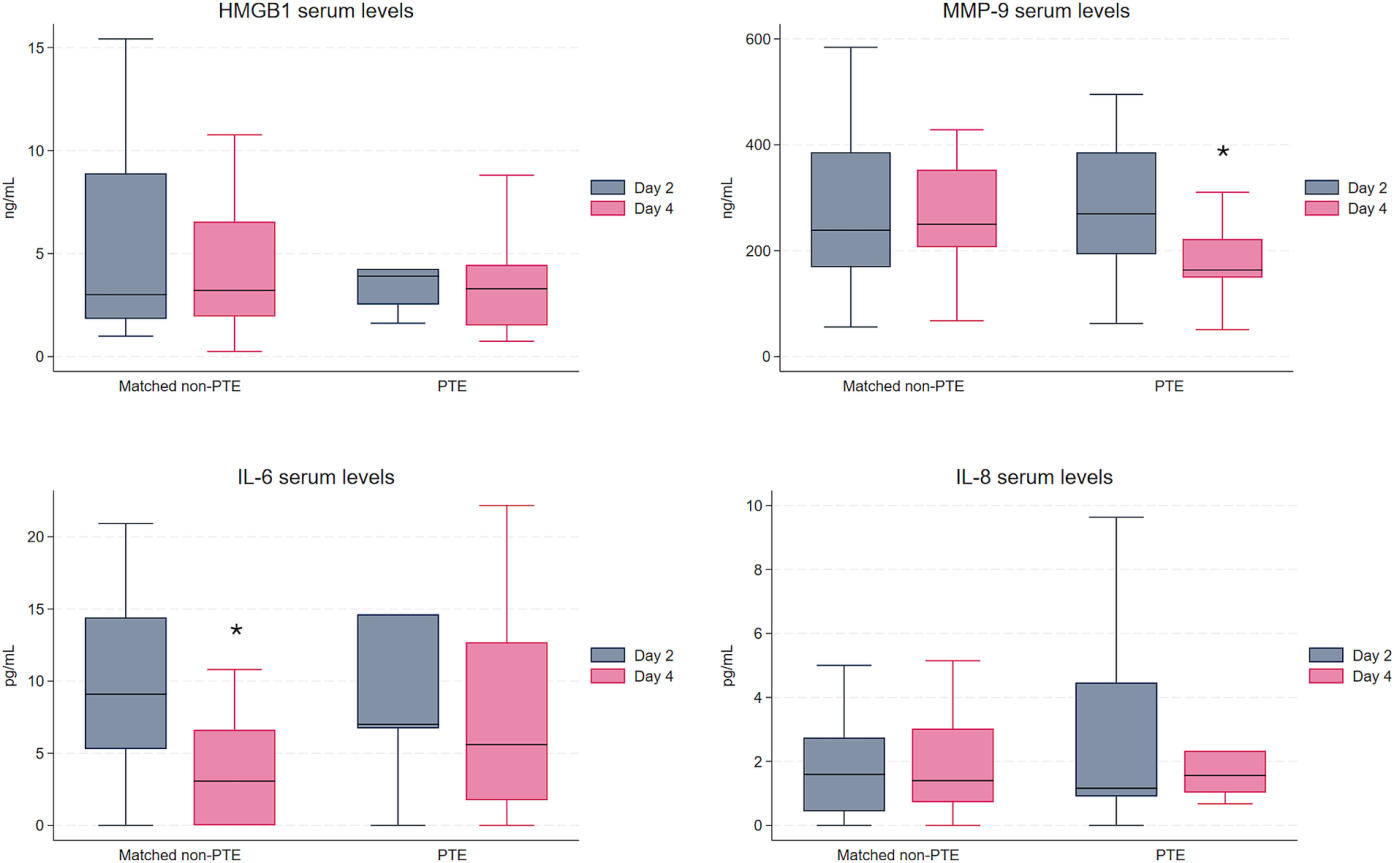
Serum levels of HMGB1, MMP-9, IL-6, and IL-8 in the PTE group (*n* = 13) and the matched non-PTE group (*n* = 26). To enhance visualization, outliers are not displayed; they were included in all analyses. *Indicates a statistically significant change between day 2 and day 4 within the group. HMGB1 and MMP-9 in ng/mL, IL-6 and IL-8 in pg/mL. HMGB1, High Mobility Group Box 1; MMP-9, Matrix metalloproteinase 9; IL-6, Interleukin-6; IL-8, Interleukin-8.

Comparison of the PTE group with the matched non-PTE group (*n* = 26) showed similar results to those of the total cohort. There were no statistically significant differences in median concentration of any of the proteins at any of the time points, nor in the change from day 2 to day 4 between the PTE group and the matched non-PTE group. As in the total non-PTE group, median IL-6 level decreased over time within the matched non-PTE group (*p* = 0.006), while there was no decrease in the PTE group (Figure 2). At variance with the findings in the PTE and the total non-PTE group, there was no statistically significant decrease in MMP-9 between the two time points in the matched non-PTE group. Changes in HMGB1 and IL-8 levels were not statistically significant within groups.

#### 3.2.3 HMGB1, MMP-9, IL-6 and IL-8 and long-term mortality

Median levels of IL-6 at day 2 and day 4 were higher in patients who died during follow-up (*n* = 6, non-PTE), as shown in Table 3. In particular, survivors exhibited significantly lower median level of IL-6 on day 4 (*p* = 0.03). The levels of the other proteins were not significantly different between the groups at either timepoint (Table 3) or in the change from day 2 to day 4. Those who died were older (70 vs. 47 years old, *p* = 0.01), but we found no association between age and IL-6 levels when stratifying the whole group by age (≥65 vs. < 65 years of age, *p* = 0.84).

## 4 Discussion

In this prospective multicenter study, we did not find significant differences in serum levels of the inflammatory mediators HMGB1, MMP-9, IL-6 and IL-8 between TBI patients who developed PTE and those who did not, implying that they may not serve as sensitive early biomarkers of PTE prediction.

Notably, our findings show a significant decline in IL-6 levels over time in TBI patients who did not develop PTE, whereas no similar decline was observed in those who did, suggesting a prolonged inflammatory state in patients who develop PTE. This evidence supports the fact that, although inflammation is a homeostatic response to brain injury triggered to promote tissue repair and recovery, when excessive or prolonged it can be harmful and contribute to complications after TBI, including epilepsy (13, 14).

At variance with our findings, a study in Indian patients with moderate to severe TBI reported significantly higher serum levels of IL-6 on admission in patients developing PTE, suggesting that IL-6 could serve as an early biomarker of PTE (35). The IL-6 levels reported were notably higher than in our study, including in the non-PTE group (median [IQR] 30.02 [9.22, 92.86] vs. 6.1 [0, 12.2]) in our study (35). The discrepancy could relate to timing of sampling or to sample collection and assay modality. Accurate detection of cytokines is challenging due to their low concentrations, short half-life and potential interference from factors present in blood (24). This could possibly also explain the low IL-8 levels, which did not differ between PTE and non-PTE groups, and undetectable IL-10 in our study. A median level of both cytokines has previously been found to be ~17 pg/mL in healthy controls (36).

Consistent with previous reports, we found that high levels of IL-6 are associated with long-term mortality after TBI, supporting that serum IL-6 may be of value as a prognostic biomarker of long-term mortality post-TBI (36, 37). However, the broad IQR in our results indicates substantial variability in the measurements, which may impact the clinical utility of this measure.

A recent Malaysian study of patients with mild to severe TBI reported that serum levels of HMGB1 were elevated 12 months post-TBI in patients who developed PTE (38). Similar to our results, that study found no difference in serum levels in the acute phase after TBI between PTE and non-PTE patients, indicating that HMGB1 may not be a reliable early biomarker of epileptogenesis after TBI in humans. Contrary to a previous study on patients with TBI, we did not find any association between HMGB1 levels and mortality (39). In our analyses, only patients that survived the first 3 months were included, suggesting that HMGB1 may be a better predictor of mortality in the acute phase after TBI.

Our results show that serum MMP-9 is substantially elevated during the first days after severe TBI compared to blood levels in healthy controls [>200 ng/mL vs. 41.52 ± 15.13 (40)]. We observed an initial increase and a subsequent decrease in blood MMP-9 post-TBI as described by other groups (41, 42). We did not find any difference in MMP-9 levels between PTE and non-PTE groups. Increased expression of MMP-9 has previously been found in epileptogenic brain tissue of patients after status epilepticus, in patients with drug-resistant epilepsy and in a post-SE rat model of epileptogenesis linking MMP-9 to epileptogenesis (27). However, in an animal model of TBI, changes in brain tissue levels of MMP-9 were not reflected in serum, suggesting that blood MMP-9 may not be a reliable marker of post-TBI epileptogenesis (28). In contrast to what we expected, we observed a significant reduction of serum MMP-9 from day 2 to day 4 in the PTE group, but not in the matched non-PTE group. MMP-9 is expressed and released by various cell types, including neurons. It could be hypothesized that massive and widespread neuronal death, such as that observed in the most severe TBIs, could lead to greater neuronal loss and consequently greater reduction in MMP-9 production. This could explain the more pronounced decrease in MMP-9 levels in the PTE patients who presumably have the most severe brain damage. It is worth investigating whether a faster decline in MMP-9 may correlate with increased markers of brain injury severity such as glial fibrillary acidic protein (GFAP) and ubiquitin C-terminal hydrolase L1 (UCH-L1).

## 5 Limitations and strength

A possible association between the levels of inflammatory markers and PTE might have been missed due to the small sample size, particularly the low number of PTE cases. The proportion of patients who developed PTE was half of that expected based on previous epidemiological, observational and placebo-controlled interventional studies, although more in line with a recent registry-based study from Norway (1, 8, 29, 43–45). Despite strict inclusion criteria, the heterogeneity of the TBI population in terms of medical history, injury severity, concomitant injuries, and acute treatments may influence peripheral levels of inflammatory markers and complicate the assessment of their utility as prognostic biomarkers (46). Notably, penetrating injury and multitrauma injury were more prevalent in the PTE group in our study, which might have introduced bias into our results. Due to the small size of the PTE group (*n* = 13) and large variability in biomarker levels, performing a regression analysis to adjust for these characteristics would likely yield unreliable results.

In spite of our study limitations, the prospective design and the long-term clinical follow-up of the patients strengthen the study. The multicenter approach enhances the generalizability of the results, although it opens up the possibility of variations in data collection that may affect data quality. In the US, management of moderately severe and severe TBI follows accepted guidelines (31). Clinical management of TBI is broadly similar between US and European trauma centers, but the study did not include standardization of clinical management, and it is possible that there may have been undocumented differences in the management between US and European centers. The limitations of our study highlight the challenges in the search for biomarkers of human epileptogenesis. The processes involved in epileptogenesis after TBI are complex, and larger studies with longitudinal and repeated blood sampling are needed to better understand the dynamic changes in inflammatory mediators and their reliability as prognostic biomarkers of PTE. The risk of high early attrition in study populations with severe TBI and the potential for a lower-than-expected rate of PTE must be accounted for when planning future studies.

## 6 Conclusion

In this prospective study of patients with TBI and anticipated high PTE risk, early post-injury measurements of the inflammatory mediators HMGB1, MMP-9, IL-6, IL-8 and IL-10 did not predict PTE. Our findings show a more rapid decline in IL-6 levels in the non-PTE group, suggesting a faster resolution of inflammation in patients who do not develop PTE, supporting the role of neuroinflammatory mechanisms in epileptogenesis. Further, serum IL-6 can be of value as a prognostic biomarker of long-term mortality post-TBI. The study highlights the need for collaborative efforts with larger cohorts to facilitate biomarker discovery and validation and ultimately improve management of PTE.

## Data Availability

The datasets presented in this article are not readily available because they include patient-sensitive information that falls under personal data protection regulations. Requests to access the datasets should be directed to the principal investigator Pavel Klein, kleinp@epilepsydc.com.

## References

[1] Annegers JF Hauser WA Coan SP Rocca WA. A population-based study of seizures after traumatic brain injuries. N Engl J Med. (1998) 338:20–4. doi: 10.1056/NEJM199801013380104, PMID: 9414327

[2] Christensen J Pedersen MG Pedersen CB Sidenius P Olsen J Vestergaard M. Long-term risk of epilepsy after traumatic brain injury in children and young adults: a population-based cohort study. Lancet. (2009) 373:1105–10. doi: 10.1016/S0140-6736(09)60214-2, PMID: 19233461

[3] Karlander M Ljungqvist J Sörbo A Zelano J. Risk and cause of death in post-traumatic epilepsy: a register-based retrospective cohort study. J Neurol. (2022) 269:6014–20. doi: 10.1007/s00415-022-11279-5, PMID: 35852600 PMC9553825

[4] Burke J Gugger J Ding K Kim JA Foreman B Yue JK . Association of posttraumatic epilepsy with 1-year outcomes after traumatic brain injury. JAMA Netw Open. (2021) 4:e2140191. doi: 10.1001/jamanetworkopen.2021.40191, PMID: 34964854 PMC8717106

[5] Strzelczyk A Aledo-Serrano A Coppola A Didelot A Bates E Sainz-Fuertes R . The impact of epilepsy on quality of life: findings from a European survey. Epilepsy Behav. (2023) 142:109179. doi: 10.1016/j.yebeh.2023.109179, PMID: 37058861

[6] Klein P Dingledine R Aronica E Bernard C Blumcke I Boison D . Commonalities in epileptogenic processes from different acute brain insults: do they translate? Epilepsia. (2018) 59:37–66. doi: 10.1111/epi.13965, PMID: 29247482 PMC5993212

[7] Pitkänen A Lukasiuk K Dudek FE Staley KJ. Epileptogenesis. Cold Spring Harb Perspect Med. (2015) 5:a022822. doi: 10.1101/cshperspect.a022822, PMID: 26385090 PMC4588129

[8] Klein P Tyrlikova I. No prevention or cure of epilepsy as yet. Neuropharmacology. (2020) 168:107762. doi: 10.1016/j.neuropharm.2019.107762, PMID: 31499048

[9] Klein P Friedman A Hameed MQ Kaminski RM Bar-Klein G Klitgaard H . Repurposed molecules for Antiepileptogenesis: missing an opportunity to prevent epilepsy? Epilepsia. (2020) 61:359–86. doi: 10.1111/epi.16450, PMID: 32196665 PMC8317585

[10] Klein P Koepp M Rotenberg A Hameed MQ Löscher W. Clinical trials of prevention of acquired epilepsy: new proof-of-concept approach to restart trials. Epilepsia. (2025) 66:2679–89. doi: 10.1111/epi.18394, PMID: 40184261

[11] Pitkänen A Paananen T Kyyriäinen J Das Gupta S Heiskanen M Vuokila N . Biomarkers for posttraumatic epilepsy. Epilepsy Behav. (2021) 121:107080. doi: 10.1016/j.yebeh.2020.107080, PMID: 32317161

[12] Bruckhaus AA Asifriyaz T Kriukova K O'Brien TJ Agoston DV Staba RJ . Exploring multimodal biomarker candidates of post-traumatic epilepsy following moderate to severe traumatic brain injury: a systematic review and meta-analysis. Epilepsia. (2025) 66:6–32. doi: 10.1111/epi.18131, PMID: 39530841 PMC13730297

[13] Webster KM Sun M Crack P O'Brien TJ Shultz SR Semple BD. Inflammation in epileptogenesis after traumatic brain injury. J Neuroinflammation. (2017) 14:10. doi: 10.1186/s12974-016-0786-1, PMID: 28086980 PMC5237206

[14] Vezzani A Balosso S Ravizza T. Neuroinflammatory pathways as treatment targets and biomarkers in epilepsy. Nat Rev Neurol. (2019) 15:459–72. doi: 10.1038/s41582-019-0217-x, PMID: 31263255

[15] Vezzani A Maroso M Balosso S Sanchez MA Bartfai T. Il-1 receptor/toll-like receptor signaling in infection, inflammation, stress and neurodegeneration couples Hyperexcitability and seizures. Brain Behav Immun. (2011) 25:1281–9. doi: 10.1016/j.bbi.2011.03.018, PMID: 21473909

[16] Villasana-Salazar B Vezzani A. Neuroinflammation microenvironment sharpens seizure circuit. Neurobiol Dis. (2023) 178:106027. doi: 10.1016/j.nbd.2023.106027, PMID: 36736598

[17] Morganti-Kossmann MC Rancan M Stahel PF Kossmann T. Inflammatory response in acute traumatic brain injury: a double-edged sword. Curr Opin Crit Care. (2002) 8:101–5. doi: 10.1097/00075198-200204000-00002, PMID: 12386508

[18] Das M Mohapatra S Mohapatra SS. New perspectives on central and peripheral immune responses to acute traumatic brain injury. J Neuroinflammation. (2012) 9:236. doi: 10.1186/1742-2094-9-236, PMID: 23061919 PMC3526406

[19] Ravizza T Terrone G Salamone A Frigerio F Balosso S Antoine DJ . High mobility group box 1 is a novel pathogenic factor and a mechanistic biomarker for epilepsy. Brain Behav Immun. (2018) 72:14–21. doi: 10.1016/j.bbi.2017.10.008, PMID: 29031614

[20] Pauletti A Terrone G Shekh-Ahmad T Salamone A Ravizza T Rizzi M . Targeting oxidative stress improves disease outcomes in a rat model of acquired epilepsy. Brain. (2019) 142:e39. doi: 10.1093/brain/awz130, PMID: 31145451 PMC6598637

[21] Walker LE Sills GJ Jorgensen A Alapirtti T Peltola J Brodie MJ . High-mobility group box 1 as a predictive biomarker for drug-resistant epilepsy: a proof-of-concept study. Epilepsia. (2022) 63:e1–6. doi: 10.1111/epi.17116, PMID: 34747496

[22] Kan M Song L Zhang X Zhang J Fang P. Circulating high mobility group box-1 and toll-like receptor 4 expressions increase the risk and severity of epilepsy. Braz J Med Biol Res. (2019) 52:e7374. doi: 10.1590/1414-431x20197374, PMID: 31241711 PMC6596364

[23] Gallentine WB Shinnar S Hesdorffer DC Epstein L Nordli DR Jr Lewis DV . Plasma cytokines associated with febrile status epilepticus in children: a potential biomarker for acute hippocampal injury. Epilepsia. (2017) 58:1102–11. doi: 10.1111/epi.13750, PMID: 28448686 PMC5482499

[24] Liu C Chu D Kalantar-Zadeh K George J Young HA Liu G. Cytokines: from clinical significance to quantification. Adv Sci (Weinh). (2021) 8:e2004433. doi: 10.1002/advs.202004433, PMID: 34114369 PMC8336501

[25] Ruffolo G Alfano V Romagnolo A Zimmer T Mills JD Cifelli P . Gaba(a) receptor function is enhanced by Interleukin-10 in human epileptogenic Gangliogliomas and its effect is counteracted by interleukin-1β. Sci Rep. (2022) 12:17956. doi: 10.1038/s41598-022-22806-9, PMID: 36289354 PMC9605959

[26] Lima R Simon D Silva W Nabinger DD Regner A. Prognostic utility of early plasma matrix metalloproteinases -2 and -9 concentrations after severe traumatic brain injury. Rev Bras Ter Intensiva. (2020) 32:418–25. doi: 10.5935/0103-507x.20200071, PMID: 33053032 PMC7595721

[27] Broekaart DW Bertran A Jia S Korotkov A Senkov O Bongaarts A . The matrix metalloproteinase inhibitor Ipr-179 has antiseizure and antiepileptogenic effects. J Clin Invest. (2021) 131:e138332. doi: 10.1172/jci138332, PMID: 33141761 PMC7773344

[28] Pijet B Stefaniuk M Kostrzewska-Ksiezyk A Tsilibary PE Tzinia A Kaczmarek L. Elevation of Mmp-9 levels promotes epileptogenesis after traumatic brain injury. Mol Neurobiol. (2018) 55:9294–306. doi: 10.1007/s12035-018-1061-5, PMID: 29667129 PMC6208832

[29] Englander J Bushnik T Duong TT Cifu DX Zafonte R Wright J . Analyzing risk factors for late posttraumatic seizures: a prospective, multicenter investigation. Arch Phys Med Rehabil. (2003) 84:365–73. doi: 10.1053/apmr.2003.50022, PMID: 12638104

[30] Temkin NR. Risk factors for posttraumatic seizures in adults. Epilepsia. (2003) 44:18–20. doi: 10.1046/j.1528-1157.44.s10.6.x, PMID: 14511390

[31] Brain Trauma Foundation. Guidelines for the management of severe Tbi, 4th Edition (2016). Available online at: https://braintrauma.org/coma/guidelines/guidelines-for-the-management-of-severe-tbi-4th-ed

[32] Harris PA Taylor R Thielke R Payne J Gonzalez N Conde JG. Research electronic data capture (Redcap)--a metadata-driven methodology and workflow process for providing translational research informatics support. J Biomed Inform. (2009) 42:377–81. doi: 10.1016/j.jbi.2008.08.010, PMID: 18929686 PMC2700030

[33] Lehner J Wittwer C Fersching D Siegele B Holdenrieder S Stoetzer OJ. Methodological and preanalytical evaluation of an Hmgb1 immunoassay. Anticancer Res. (2012) 32:2059–62. PMID: 22593488

[34] Pasetto L Grassano M Pozzi S Luotti S Sammali E Migazzi A . Defective cyclophilin a induces Tdp-43 proteinopathy: implications for amyotrophic lateral sclerosis and frontotemporal dementia. Brain. (2021) 144:3710–26. doi: 10.1093/brain/awab333, PMID: 34972208 PMC8719849

[35] Choudhary A Varshney R Kumar A Kaushik K. A prospective study of novel therapeutic targets interleukin 6, tumor necrosis factor Α, and interferon Γ as predictive biomarkers for the development of posttraumatic epilepsy. World Neurosurg X. (2021) 12:100107. doi: 10.1016/j.wnsx.2021.100107, PMID: 34195601 PMC8233159

[36] Tsitsipanis C Miliaraki M Paflioti E Lazarioti S Moustakis N Ntotsikas K . Inflammation biomarkers Il-6 and Il-10 may improve the diagnostic and prognostic accuracy of currently authorized traumatic brain injury tools. Exp Ther Med. (2023) 26:364. doi: 10.3892/etm.2023.12063, PMID: 37408863 PMC10318605

[37] Raheja A Sinha S Samson N Bhoi S Subramanian A Sharma P . Serum biomarkers as predictors of long-term outcome in severe traumatic brain injury: analysis from a randomized placebo-controlled phase II clinical trial. J Neurosurg. (2016) 125:631–41. doi: 10.3171/2015.6.Jns15674, PMID: 26722854

[38] Ngadimon IW Mohan D Shaikh MF Khoo CS Tan HJ Lee YM . Hmgb1 blood levels and neurological outcomes after traumatic brain injury: insights from an exploratory study. Epilepsia Open. (2025) 10:494–507. doi: 10.1002/epi4.70001, PMID: 39937590 PMC12014938

[39] Wang KY Yu GF Zhang ZY Huang Q Dong XQ. Plasma high-mobility group box 1 levels and prediction of outcome in patients with traumatic brain injury. Clin Chim Acta. (2012) 413:1737–41. doi: 10.1016/j.cca.2012.07.002, PMID: 22789964

[40] Li Y Han X Luo S Huang H Huang X Li M . Predictive value of longitudinal changes of serum matrix metalloproteinase-9 and brain-derived neurotrophic factor in acute ischemic stroke. Front Aging Neurosci. (2022) 14:952038. doi: 10.3389/fnagi.2022.952038, PMID: 36092813 PMC9452807

[41] Suehiro E Fujisawa H Akimura T Ishihara H Kajiwara K Kato S . Increased matrix metalloproteinase-9 in blood in association with activation of interleukin-6 after traumatic brain injury: influence of hypothermic therapy. J Neurotrauma. (2004) 21:1706–11. doi: 10.1089/neu.2004.21.1706, PMID: 15684762

[42] Vilalta A Sahuquillo J Rosell A Poca MA Riveiro M Montaner J. Moderate and severe traumatic brain injury induce early overexpression of systemic and brain gelatinases. Intensive Care Med. (2008) 34:1384–92. doi: 10.1007/s00134-008-1056-1, PMID: 18350273

[43] Temkin NR Dikmen SS Anderson GD Wilensky AJ Holmes MD Cohen W . Valproate therapy for prevention of posttraumatic seizures: a randomized trial. J Neurosurg. (1999) 91:593–600. doi: 10.3171/jns.1999.91.4.0593, PMID: 10507380

[44] Temkin NR Dikmen SS Wilensky AJ Keihm J Chabal S Winn HR. A randomized, double-blind study of phenytoin for the prevention of post-traumatic seizures. N Engl J Med. (1990) 323:497–502. doi: 10.1056/NEJM199008233230801, PMID: 2115976

[45] Sødal HF Nordseth T Rasmussen AJO Rosseland LA Stenehjem JS Gran JM . Risk of epilepsy after traumatic brain injury: a Nationwide Norwegian matched cohort study. Front Neurol. (2024) 15:1411692. doi: 10.3389/fneur.2024.1411692, PMID: 38903174 PMC11188468

[46] Saletti PG Mowrey WB Liu W Li Q McCullough J Aniceto R . Early preclinical plasma protein biomarkers of brain trauma are influenced by early seizures and Levetiracetam. Epilepsia Open. (2023) 8:586–608. doi: 10.1002/epi4.12738, PMID: 37026764 PMC10235584

